# Caseload and Case Fatality of Lassa Fever in Nigeria, 2001–2018: A Specialist Center's Experience and Its Implications

**DOI:** 10.3389/fpubh.2019.00170

**Published:** 2019-06-25

**Authors:** George O. Akpede, Danny A. Asogun, Sylvanus A. Okogbenin, Simeon O. Dawodu, Mojeed O. Momoh, Andrew E. Dongo, Chiedozie Ike, Ekaete Tobin, Nosa Akpede, Ephraim Ogbaini-Emovon, Adetunji E. Adewale, Oboratare Ochei, Frank Onyeke, Martha O. Okonofua, Rebecca O. Atafo, Ikponmwosa Odia, Donatus I. Adomeh, George Odigie, Caroline Ogbeifun, Ekene Muoebonam, Chikwe Ihekweazu, Michael Ramharter, Andres Colubri, Pardis C. Sabeti, Christian T. Happi, Stephan Günther, Dennis E. Agbonlahor

**Affiliations:** ^1^Institute of Lassa Fever Research and Control, Irrua Specialist Teaching Hospital, Irrua, Nigeria; ^2^Department of Paediatrics, Irrua Specialist Teaching Hospital, Irrua, Nigeria; ^3^Department of Community Medicine, Irrua Specialist Teaching Hospital, Irrua, Nigeria; ^4^Department of Obstetrics and Gynaecology, Irrua Specialist Teaching Hospital, Irrua, Nigeria; ^5^Department of Surgery, Irrua Specialist Teaching Hospital, Irrua, Nigeria; ^6^Department of Nursing Services, Irrua Specialist Teaching Hospital, Irrua, Nigeria; ^7^Department of Medical Records, Irrua Specialist Teaching Hospital, Irrua, Nigeria; ^8^Nigeria Centre for Disease Control, Abuja, Nigeria; ^9^Department of Tropical Medicine, Bernhard Nocht Institute for Tropical Medicine & I. Department of Medicine University Medical Center Hamburg-Eppendorf, Hamburg, Germany; ^10^Department of Organismic and Evolutionary Biology, Harvard University, Cambridge, MA, United States; ^11^Department of Biological Sciences and African Center of Excellence for Genomics of Infectious Diseases, Redeemer's University, Ede, Nigeria; ^12^Department of Virology, Bernhard Nocht Institute for Tropical Medicine, Hamburg, Germany; ^13^Department of Microbiology, Ambrose Alli University, Ekpoma, Nigeria

**Keywords:** case fatality, caseload, center's experience, implications, Lassa fever, outbreaks, Nigeria, trends

## Abstract

**Background:** The general lack of comprehensive data on the trends of Lassa fever (LF) outbreaks contrasts with its widespread occurrence in West Africa and is an important constraint in the design of effective control measures. We reviewed the contribution of LF to admissions and mortality among hospitalized patients from 2001 to 2018 in the bid to address this gap.

**Methods:** Observational study of LF caseload and mortality from 2001 to 18 in terms of the contribution of confirmed LF to admissions and deaths, and case fatality (CF) among patients with confirmed LF at a specialist center in Nigeria. The diagnosis of LF was confirmed using reverse transcription polymerase chain reaction (RT-PCR) test, and medians and frequencies were compared using Kruskal-Wallis, Mann-Whitney and χ2 tests, with *p*-values <0.05 taken as significant.

**Results:** The contribution of confirmed LF to deaths (362/9057, 4.0%) was significantly higher than to admissions (1,298/185,707, 0.7%; OR [95% CI] = 5.9 [5.3, 6.7], *p* < 0.001). The average CF among patients with confirmed LF declined from 154/355 (43%) in 2001–09 to 183/867 (21.1%) (OR [95% CI] = 2.9 [2.2, 3.7], *p* < 0.001) in 2011–18. The annual CF declined from 94% in 2001 to 15% in 2018 whereas the caseload increased from 0.3 to 3.4%. The outbreaks were characterized by irregular cycles of high caseload in 2005–2007, 2012–2014, and 2016–2018, and progressive blurring of the seasonality.

**Conclusion:** LF outbreaks in Nigeria have upgraded spatially and temporally, with the potential for cycles of increasing severity. The strategic establishment of LF surveillance and clinical case management centers could be a pragmatic and cost-effective approach to mitigating the outbreaks, particularly in reducing the associated CF. Urgent efforts are needed in reinvigorating extant control measures while the search for sustainable solutions continues.

## Introduction

Lassa fever (LF), one of the severe viral hemorrhagic fevers (VHF), was first reported from Nigeria in 1969 (1). Since then, there have been repeated outbreaks in Nigeria (2) and other West African countries (3, 4), particularly Sierra Leone (5) and Liberia (6) but also Togo (4), and the Republic of Benin (4). In addition, LF is the most exported VHF, particularly to Europe (4, 7, 8) and America (4, 8–10).

The control of LF has been neglected (2), even within the context of neglected tropical diseases. Nonetheless, one of the foremost priorities should be how to reduce the high hospital case fatality (2, 6, 11), which has remained relatively high despite the availability of an arguably effective therapeutic agent, ribavirin, for several decades (12, 13). While many factors could account for the high case fatality, including late presentation (12) and other delays in diagnosis (14), the dearth of human and material resources for diagnosis and treatment may be important but somewhat overlooked factors. The place of adequate case management in the outcome of such severe VHFs as Ebola and Marburg is attested to by North-South (15) and even intra-country South-South (16) variations in outcome.

In the bid to improve the diagnosis and outcome of VHFs including LF in developing countries, the World Health Organization (WHO) has recommended the establishment of Centers of Excellence (COE) (17). Thus, far, however, there have been only a few of such centers in West Africa, a sub region with a high endemicity and rather widespread occurrence of LF outbreaks (18–20). Among them is the LF Center at Kenema General Hospital, Sierra Leone (21) and the Institute of Lassa Fever Research and Control, ILFR&C, Irrua Specialist Teaching Hospital (ISTH), Nigeria (22). Both have been actively engaged in the management of patients with LF for many years in spite of the inherent challenges.

ISTH was designated a COE for the control of LF by the Federal Government of Nigeria in 2002. However, the subsisting situation before 2008 was far from that of a COE: Blood samples had to be sent to Europe and South Africa among others for confirmatory tests, and the results were usually not available for weeks to months; Patients with suspected LF were admitted in improvised isolation rooms or bays within the general wards, with attendant high risk of nosocomial transmission and workplace infection of staff; And, the supply of ribavirin, the agent primarily used for the treatment of LF cases, was erratic. This scenario was not good either for case management or the prevention of nosocomial transmission, nor the institution of public health measures. The ILFR&C was therefore established by the Board of Management of ISTH in 2007 as “an aggregate indigenous response to comprehensively address the LF epidemic” in seeking to address these and other challenges related to LF control in the pursuit of the hospital's mandate as a COE.

Among others, the activities of the Institute include surveillance, clinical virology, clinical case management, community engagement, outreach and enlightenment campaigns, and research. To carry out these activities, the Institute has a robust staff compliment inclusive of public health physicians, clinical virologists, nurses, biomedical scientists, medical laboratory scientists, community health extension workers, and clinicians. It also has research and training collaborations with Bernhard-Nocht Institute for Tropical Medicine (BNITM), Hamburg, Germany, Harvard and Tulane Universities, USA, and Public Health, England since 2007, 2007, and 2012, respectively. In addition, the Institute has established a close working relationship with the Nigeria Centre for Disease Control, NCDC.

**Figure 1:**
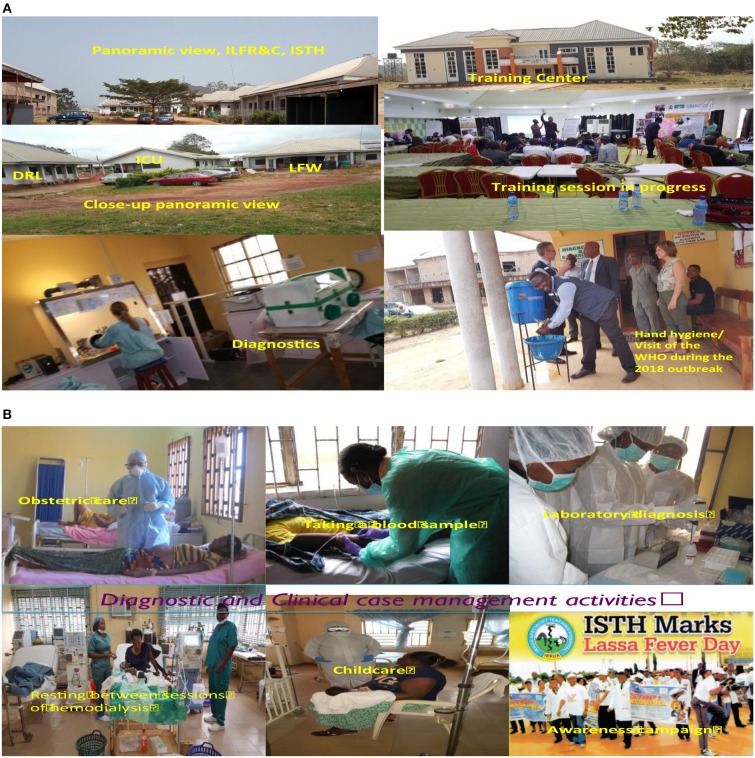
Panoramic view of the Institute of Lassa Fever Research and Control (ILFR&C), Irrua Specialist Teaching Hospital, Nigeria and its activities. **(A)** Panoramic view of the Institute, diagnostic activities, Training Center and training activities, and hand washing stand. **(B)** Panoramic view of clinical case management and diagnostic activities, and awareness campaign match on a national Lassa fever day.

A biosafety level-2+ Diagnostic and Research Laboratory (D&RL) that started operation in September 2008 is available at the Institute. There is also a 35-bedded purpose-built Lassa Fever Ward (LFW), with a dedicated Lassa fever Intensive Care Unit (LFICU), which was opened in September 2010. The LFW has sections for adult males and females including pregnant women, and children while the LFICU has a hemodialysis unit that has been in operation since 2011. A composite panoramic view of the ILFR&C including diagnostic activities in the D&RL, the LFW, hemodialysis unit, and potshots from one of the visits of the WHO during 2018 outbreak and a training session are shown in Figures 1A,B.

The first onsite laboratory confirmation of the diagnosis of LF in ISTH was made in the D&RL on September 27th, 2008 while about 1,300 patients with confirmed LF were treated in the hospital between January 2001 and September 2018. The cost of confirmatory tests for LF has been free as part of on-going research programs as well as support from NCDC, while the Federal Government of Nigeria provides Ribavirin and other care commodities such as personal protective equipment without charge for the treatment of patients.

After almost two decades since the designation of ISTH as a COE, over a decade of establishment of the Institute, and about 8 years of operation of the LFW, we thought that it might be helpful to broadly review our experiences in surveillance and clinical case management. Specifically with the view to sharing our experience in the diagnosis and treatment of LF, we sought first to review the temporal and spatial variations of the caseload of LF as indices of the trend of LF outbreaks in Nigeria. Second, we sought to describe the trends in case fatality pre- and post-commissioning of the LFW as an index of the potential impact of dedicated treatment facilities. A third perspective was to describe the determinants of hospital outcome of LF cases as seen in our center. Nigeria stands out among LF endemic countries due to her large population and landmass, widespread nature of the outbreaks, and diversity of the Lassa virus (LASV) lineages in circulation (23, 24). In addition, the outcome of LF had usually been worse in Nigeria compared to other West African countries (6). It could therefore be expected that the experiences gained in the mitigation of LF in Nigeria may be of significance in the mitigation of LF in West Africa in general.

## Subjects and Methods

The study was carried out at the ILFR&C, ISTH, Irrua, Edo State, Nigeria.

### Ethical Considerations

The study was granted exemption from ethical review by the Research and Ethics Committee of ISTH being one of service evaluation, which “is exempt from ethical review according to the National Code of Health Research Ethics, National Health Research Ethics Committee, Federal Ministry of Health, Nigeria” (22). Neither the diagnosis of LF nor treatment with ribavirin or any of the other drugs or treatment modalities was experimental in nature but were part of regular clinical practice and the data in the manuscript were drawn from available records so generated. This paper is based on de-identified data on patients evaluated and/or treated for LF in ISTH since the inception of the Lassa fever control program of the hospital.

### Methodology

Data on suspected cases and the results of confirmatory tests were retrieved from the electronic worksheet maintained at the ILFR&C. This contains information on the socio-demographic characteristics, clinical features, and results of laboratory tests carried out on patients. Data on admissions and deaths for the period 2011–2018 were retrieved from the ward register maintained in the LFW. The corresponding data on admissions and discharges of confirmed cases for the period 2001–2010 were obtained from the Medical Records Department of our hospital.

The diagnosis of LF was confirmed using Lassa virus-reverse transcription-polymerase chain reaction (LASV-RT-PCR) tests carried out on blood samples drawn from patients with suspected infections. The indications for testing and the procedures involved have been described fully previously (22, 25); for ease of reference, the indications included (22): fever ≥38°C for ≥2/7; typhoid fever and malaria excluded or just 1+ on thick smear; presence of ≥1 signs/symptoms of chest pain, sore throat, headache, muscle pain, vomiting, diarrhea; fever with bleeding or facial edema; fever unresponsive to antimalarial or antibiotic drugs >2/7 of treatment; or fever plus history of contact with confirmed Lassa fever patient in the past 3 weeks. Before establishment of the D&RL in 2008, blood samples from patients with suspected LF were sent overseas for confirmatory testing using the methods (complement fixation test and enzyme linked immunosorbent assay) described elsewhere (26, 27).

### Clinical Case Management of Patients Admitted to the LF Ward

Team leads, Medical, Pediatric, Obstetric, Nursing care, Laboratory services/IPC, and LF Diagnostic Services, supervise the management of patients in the LFW. Currently, the usual bundle of treatment includes intravenous fluids at maintenance rate, intravenous ribavirin given as a single dose daily (28, 29) or as divided doses daily (12, 13, 30), broad spectrum antibiotics usually intravenous ceftriaxone, intravenous antimalarial drugs usually artesunate, and supportive care. The latter include monitoring of vital signs, correction for fluid and electrolyte imbalance, cardiovascular support with bolus doses of intravenous fluids and treatment with inotropic drugs in patients with septic shock, maintenance of fluid balance and hemodialysis for patients with acute kidney injury (AKI), intravenous anticonvulsants for the control of convulsions, and monitoring of fluid and electrolyte balance. Patients with suspected acute abdomen are co-managed with the surgical team (29, 30). Pregnant women are managed based on viability of the fetus as assessed clinically and with Ultrasound Scan (USS) examination; those with non-viable fetus undergo evacuation of the uterus while those with a viable fetus are placed on intravenous ribavirin (31). Details of the individual subspecialty management are described in separate communications (25, 29–32).

However, the treatment regimen has been evolving, and the standard of care was not always the same throughout the period of the study. For example, hemodialysis was not accessible to patients with LF at ISTH until 2011. Before then, those with AKI were usually referred for dialysis at St. Nicholas Hospital, Lagos, a private hospital which as at then was the only one willing to accept infected patients for hemodialysis. As another example, recommendations for the treatment of septic shock have also been evolving.

### Data Analysis

Categorical variables were summarized as frequencies and proportions or percentages while the quarterly distributions of caseload of LF and admissions to the LFW for the years 2009–2018, being skewed, were expressed as median and interquartile range (IQR) instead of mean ± standard deviation (33). Groups were compared using n × n Chi-square (χ^2^) tests or non-parametric tests (Kruskal-Wallis and Mann-Whitney tests) as appropriate (33, 34), using Open Epi software (http://www.openepi.com); *p* < 0.05 were taken as significant.

The results are presented as overall and disaggregated based on the origin of the patients (Edo State vs. other States) and the odds ratio (95% confidence interval) (OR [95% CI]) of observing a confirmed case given the origin of the patient (Edo State vs. other States) were calculated. The OR (95% CI) for death as an adverse outcome was also calculated for the period 2001–2009 vs. 2011–2018, the study periods pre- and post-commissioning of the LFW.

## Results

### Annual Variations in Numbers of Suspected and Confirmed Cases of LF, 2008–2018

**Figure 2:**
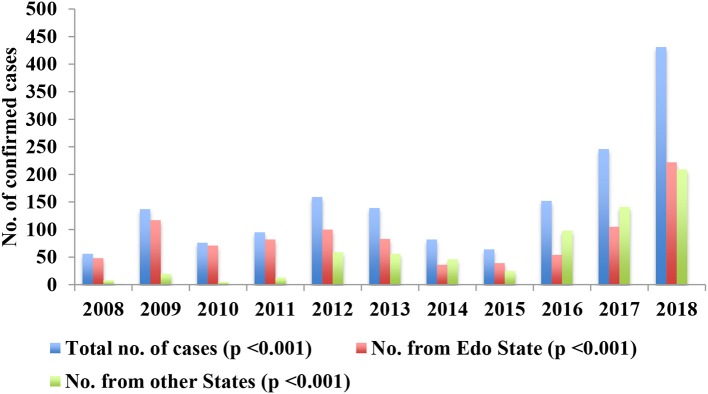
Annual variations in the numbers of confirmed cases, overall and in Edo State vs. the other States, 2008–18.

**Figure 3:**
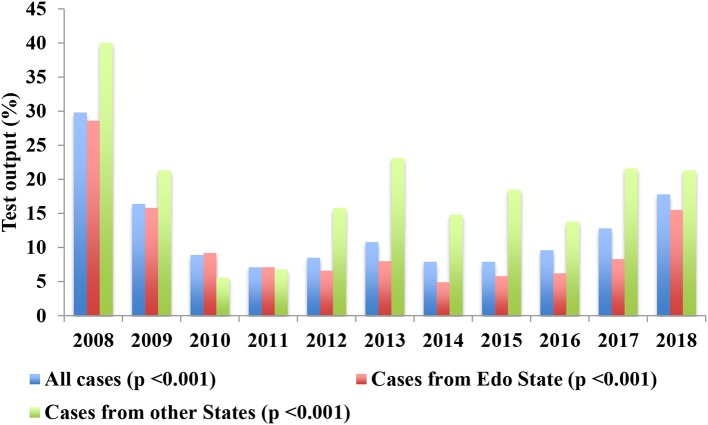
Annual variations in LASV-RT-PCR test output among suspected cases of Lassa fever, 2008–2018; % = (confirmed/suspected) × 100.

Data for 2001–08 was not available because no systematic testing of suspected cases using the criteria described under “Subjects and Methods” was undertaken over that period as we did not have facilities for testing locally then. From September 2008 to August 2018, LF was confirmed in 1,637 (11.6%) of 14,168 patients with suspected LF using LASV-RT-PCR test. The variations in annual numbers of suspected and confirmed cases are shown in Table 1 and the trend in annual numbers of confirmed cases illustrated in Figure 2 while the variations in test output from suspected cases (number of confirmed cases as a percentage of the number of suspected cases) are shown in Table 2 and illustrated in Figure 3. The variations are shown as overall and disaggregated, Edo State vs. other states.

**Table 1:** Annual variations in numbers of suspected and confirmed cases of LF, 2008–2018 and annual variations in number of geopolitical zones affected.

**Year^a^**	**Suspected cases**			**Confirmed cases**		
	**Total no**.	**n (%) from Edo State**	**No. of GPZ affected**	**Total no**.	***n* (%) from Edo State**	**No. of GPZ affected**
2008	188	168 (89.4)	1	56	48 (85.7)	1
2009	834	740 (88.7)	3	137	117 (85.4)	2
2010	858	769 (89.6)	5	76	71 (93.4)	1
2011	1,346	1,154 (85.7)	5	95	82 (86.3)	4
2012	1,887	1,514 (80.2)^#^	6	159	100 (62.9)^#^	6
2013	1,286	1,043 (81.1)^$^	6	139	83 (59.7)^$^	6
2014	1,037	729 (70.3)^+^	5	82	36 (43.9)^+^	4
2015	809	674 (83.3)^++^	6	64	39 (60.9)^++^	5
2016	1,589	877 (55.2)^+@^	6	152	54 (35.5)^+@^	6
2017	1,916	1,262 (65.9)^+&^	6	246	105 (42.7)^+&^	5
2018	2,418	1,437 (59.4)^**^	6	431	222 (51.5)^**^	5
Total	14,168	10,367 (73.2)^#&^	6	1,637	957 (58.5)^#&^	6
Av. No. of GPZ affected, 2008–11 vs. 2012-18			3.5 v. 5.9	NA	NA	2 vs. 5.3
p^***^	NA	NA (<0.001)	NA	NA	NA (<0.001)	NA

*GPZ, geopolitical zones; Av. No., average number; vs., versus; NA, Not applicable*.

a *Abuja/Federal Capital Territory had both suspected and confirmed cases in 2009, 2012, 2013, and 2018 but only suspected cases in the other years*.

Significant differences between the proportions of suspected vs confirmed cases from Edo State for each year are indicated with similar symbols as follows: p < 0.001^#, $, +, ++, +@, +&, #&^ and p = 0.003**.

*** *χ^2^ test*.

**Table 2:** Annual variations in LASV-PCR-test output among suspected cases of LF, 2008–18.

**Year**	**Total no. of confirmed/No. of suspected cases tested (%)**	**No. of confirmed/No. of suspected cases tested from Edo State (%)**	**No. of confirmed/No. of suspected cases tested from other States (%)**	**OR (95% CI)**
2008	56/188 (29.8)	48/168 (28.6)	8/20 (40)	0.6 (0.2, 1.6)
2009	137/834 (16.4)	117/740 (15.8)	20/94 (21.3)	0.7 (0.4, 1.2)
2010	76/858 (8.9)	71/769 (9.2)	5/89 (5.6)	1.7 (0.7, 4.4)
2011	95/1,346 (7.1)	82/1,154 (7.1)	13/192 (6.8)	1.1 (0.6, 1.9)
2012	159/1,887 (8.5)	100/1,514 (6.6)	59/373 (15.8)	0.4 (0.3, 0.5)^*^
2013	139/1,286 (10.8)	83/1,043 (8.0)	56/243 (23.1)	0.3 (0.2, 0.4)^*^
2014	82/1,037 (7.9)	36/729 (4.9)	46/308 (14.8)	0.3 (0.2, 0.5)^*^
2015	64/809 (7.9)	39/674 (5.8)	25/135 (18.5)	0.3 (0.2, 0.5)^*^
2016	152/1,589 (9.6)	54/877 (6.2)	98/712 (13.8)	0.4 (0.3, 0.6)^*^
2017	246/1,916 (12.8)	105/1,262 (8.3)	141/654 (21.6)	0.3 (0.3, 0.4)^*^
2018	431/2,418 (17.8)	222/1,437 (15.5)	209/981 (21.3)	0.7 (0.6, 0.8)^*^
Total	1,637/14,168 (11.6)	957/1,0367 (9.2)	680/3,801 (17.9)	0.5 (0.4, 0.5)^*^
p^***^	<0.001	<0.001	<0.001	NA

*OR (95% CI), Odds ratio (95% Confidence Interval) of test yield of suspected cases from Edo vs. other States*,

* *p < 0.001. NA, Not applicable*.

*** *χ^2^ test*.

There were mild increases in the number of confirmed cases in 2009 and 2012/13 followed by a decreases in 2010–11 and 2014/15, after which there was a steady increase from 2016 through 2017 to 2018 overall, 2017–2018 in Edo State and 2016–2018 in the other States (Figure 2). The trend of overall steady decline in the proportion of suspected (*p* < 0.001) and confirmed (*p* < 0.001) cases from Edo State was highly significant (Table 1). Edo State accounted for >85% of both suspected and confirmed cases from 2008 to 2011, 70-83% of suspected cases vs. 44–63% of confirmed cases from 2012 to 2015 and 55–66% of suspected vs. 36–52% of confirmed cases from 2016 to 2018. There were significant differences (*p* = 0.003–*p* < 0.001) between the proportions of suspected vs. confirmed cases from Edo State as a percentage of the total numbers of suspected and confirmed cases from 2012 to 2018 (Table 1).

The involvement of geopolitical zones is also shown in Table 1. Suspected cases were drawn from an average of 3.5 (58.3%) of the six geopolitical zones in 2008–11 and 5.9 (98.3%) in 20012–18 while confirmed cases were from 2 (33.3) in 2008–11 and 5.3 (88.3%) in 2012–18. There were suspected cases from Abuja and the Federal Capital Territory (FCT) every year from 2009 to 18 while confirmed cases were recorded therefrom only in 2009, 2012–13, and 2018.

The annual variations in test output are shown in Table 2 and illustrated in Figure 3.The overall test output dropped from about 30% in 2008 through 16% in 2009 to 7–11% between 2010 and 2016, and then increased to about 13% in 2017 and 18% in 2018. The output from among suspected cases from Edo State followed a similar pattern but that among patients from other states increased again much earlier from 2012 after the initial decreases from 2008 through 2009 (Figure 3). The trend in test output from 2008 to 2018 was highly significant overall (*p* < 0.001), and among cases from Edo State (*p* < 0.001) and the other States (*p* < 0.001) (Table 2). The test output among suspected cases from the other States was also significantly higher than that among cases from Edo State from 2012 to 2018 (*p* < 0.001 yearly for the difference in test output between Edo State and the other States from 2012 to 18) (Table 2).

### Annual Variations in the Contribution of Lassa Fever to Admissions and Deaths, 2001–18

Between January 2001 and September 30th, 2018, a total of 185,707 patients were admitted at ISTH, Irrua and among them 9,057 (4.9%) died. Patients with confirmed LF constituted 1,298 (0.7%) of the admissions, and 362 (4%) of the deaths (OR [95% CI] of the contribution of LF to deaths vs. admissions = 5.9 [5.3, 6.7], *p* < 0.001). The overall case fatality among patients with LF was 362/1,298 (27.9%) while that among the other patients was 8,695/184,409 (4.7%). The difference was highly significant (OR [91% CI] = 7.8 [6.9, 8.8], *p* < 0.001).

**Figure 4:**
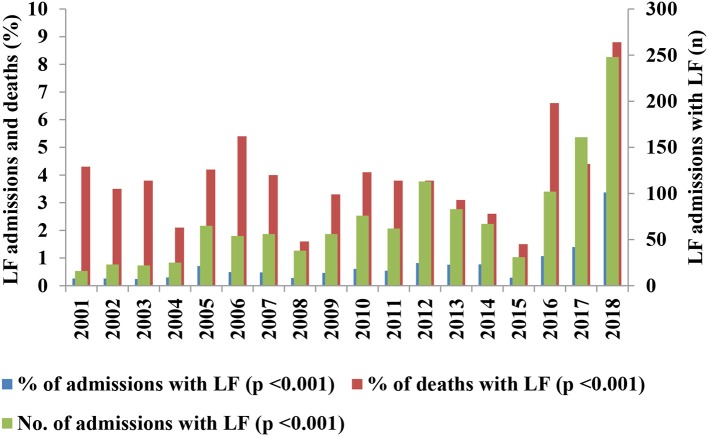
Contribution of Lassa fever to admissions and deaths, 2001–2018; % = (no. of admissions or deaths with LF/total no. of admissions or deaths) × 100.

The annual variation in contribution of LF to admissions and deaths are shown in Table 3 and illustrated in Figure 4. The contribution of LF to admissions was low (range = 0.3–0.8%) from 2001 to 2015, then rose sharply from 2016 (1.1%) through 2017 (1.4%) to 2018 (3.4%); the mean ± standard deviation of the proportion of admissions from 2001 to 15 (0.5% ± 0.2%) was significantly (*p* < 0.001) lower than that from 2016 to 18 (1.9% ± 1.2%). The overall trend was that of an increasing caseload (both in terms of the number of cases and the proportion of admissions) (*p* < 0.001), with a major surge in number and proportion of admissions with LF in 2018 (*n* = 248, 3.4% of admissions) but which began from 2016 and lesser surges in 2005 (0.7%) and 2012 (0.8%) (Figure 4). The trend of the contribution to deaths was that of irregular increases and decreases, with peaks in 2006 (5.4%), 2010 (4.1%), 2016 (6.6%), and 2018 (8.8%), and troughs in 2004 (2.1%), 2008 (1.6%), and 2015 (1.5%) (Table 3 and Figure 4). The variation in contribution of LF to death among in-patients from 2001 to 18 was highly significant (*p* < 0.001, Table 3).

**Table 3:** Contribution of LF to admissions and deaths, 2001–2018.

**Year**	**Admissions**	**Deaths**		
	**Total**	***n* (%) with LF**	**Total**	***n* (%) with LF**
2001	6,150	16 (0.3)	348	15 (4.3)
2002	8,850	23 (0.3)	426	15 (3.5)
2003	9,092	22 (0.2)	426	16 (3.8)
2004	8,388	25 (0.3)	476	10 (2.1)
2005	9,215	65 (0.7)	426	18 (4.2)
2006	11,060	54 (0.5)	520	28 (5.4)
2007	11,644	56 (0.5)	571	23 (4)
2008	13,617	38 (0.3)	636	10 (1.6)
2009	12,292	56 (0.5)	580	19 (3.3)
2010	12,374	76 (0.6)	608	25 (4.1)
2011	11,537	62 (0.5)	636	24 (3.8)
2012	13,589	113 (0.8)	662	25 (3.8)
2013	10,969	83 (0.8)	556	17 (3.1)
2014	8,671	67 (0.8)	465	12 (2.6)
2015	10,793	31 (0.3)	547	8 (1.5)
2016	9,566	102 (1.1)	488	32 (6.6)
2017	11,537	161 (1.4)	618	27 (4.4)
2018	7,363	248 (3.4)	430	38 (8.8)
Total	185,707	1,298 (0.7)	9,057	362 (4)
p^*^		<0.001		<0.001

* *χ^2^ test*.

### Seasonality of Cases and Admissions

**Figure 5:**
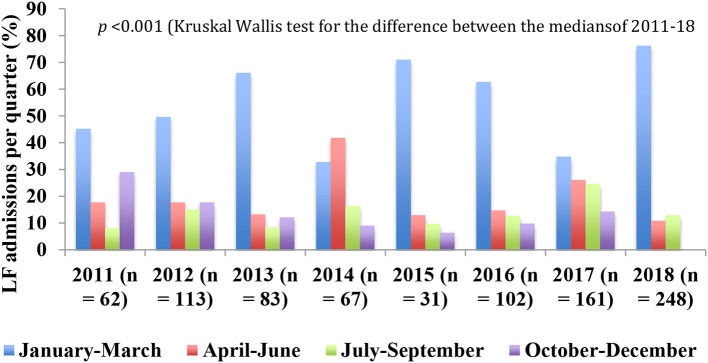
Annual variations in quarterly distribution of number of admissions to the LFW, 2011–18.

The 1st quarter of the year, January-March, is dry while the 2nd (April–June) is mostly wet or raining in the coastal and rainforest regions of the country but mostly dry in the middle belt and northern regions. The 3rd quarter (July–August) marks the peak of the raining season in the country and is usually wet or rainy throughout the country while the 4th or last quarter (October–December) is partly wet or raining in the southern regions and mostly dry in the northern regions. Overall, generally, the dry season spans November/December–April/May and the rainy season April/May–October/November. The dry season usually comprises two periods, the harmattan, which is characterized by dry, dusty weather and covers December-January/February and the heat season that spans February-March/April and is characterized by hot weather. Table 4 shows the quarterly distribution of confirmed cases from January 2009 to September 2018 and Table 5 the distribution of the number of patients with confirmed LF admitted from 2011 to 2018 while Figure 5 illustrates the quarterly trend in admissions from 2011 to 18. There was a surge in the number of cases and admissions in the first quarter of 2018 (Tables 4, 5).

**Table 4:** Quarterly distribution of confirmed cases of Lassa fever in Nigeria, 2009–2018 as seen at Irrua Specialist Teaching Hospital.

**Year**	**No. (%) of cases in**				**Total No. (%)**
	**Jan–Mar**	**Apr–Jun**	**Jul–Sept**	**Oct–Dec**	
2009	64 (46.7)	14 (10.2)	30 (21.9)	29 (21.2)	137 (100)
2010	36 (47.3)	10 (13.2)	14 (18.4)	16 (21.1)	76 (100)
2011	41 (43.2)	13 (13.7)	10 (10.5)	31 (32.6)	95 (100)
2012	82 (47.7)	39 (22.7)	23 (13.3)	28 (16.3)	172 (100)
2013	95 (68.4)	17 (12.2)	10 (7.2)	17 (12.2)	139 (100)
2014	40 (48.9)	16 (19.5)	14 (17.1)	12 (14.6)	82 (100)
2015	38 (59.7)	8 (12.5)	6 (9.4)	12 (18.7)	64 (100)
2016	113 (74.3)	13 (8.6)	9 (5.9)	17 (11.2)	152 (100)
2017	92 (37.4)	74 (30.1)	42 (17.1)	38 (18.5)	246 (100)
2018	353 (81.9)	36 (8.4)	42 (9.7)	NA	431 (100)
Total	954 (59.9)	240 (15.1)	200 (12.5)	200 (12.5)	1,594 (100)
Median (IQR)^*^^,^ ^**^	48.3 (19.4)%^#^^,^ ^@^^,^ ^&^	12.9(7.4)%^#^	11.9 (7.6)%^@^	18.5 (6.5)%^&^	

*Percentages add across. IQR, interquartile range*.

* *Median (IQR) of the proportions of cases per quarter*.

** *Kruskal-Wallis test for significance of the difference between the medians: χ^2^ = 18.5, p < 0.001*.

*Significant differences between medians are indicated by similar symbols*:

# *p < 0.001*,

@ p < 0.001, and

& *p < 0.001*.

**Table 5:** Quarterly distribution of admissions to the LF Ward, 2011–2018.

**Year**	**No. (%) admitted in**				**Total No. (%)**
	**Jan-Mar**	**Apr-Jun**	**Jul-Sep**	**Oct-Dec**	
2011	28 (45.2)	11 (17.7)	5 (8.1)	18 (29)	62 (100)
2012	56 (49.6)	20 (17.7)	17 (15)	20 (17.7)	113 (100)
2013	55 (66.3)	11 (13.2)	7 (8.4)	10 (12.1)	83 (100)
2014	22 (32.8)	28 (41.8)	11 (16.4)	6 (9)	67 (100)
2015	22 (71)	4 (12.9)	3 (9.7)	2 (6.4)	31 (100)
2016	64 (62.7)	15 (14.7)	13 (12.7)	10 (9.8)	102 (100)
2017	56 (34.8)	42 (26.1)	40 (24.5)	23 (14.3)	161 (100)
2018	189 (76.2)	27 (10.8)	32 (12.9)	NA	248 (100)
Total	**492 (56.7)**	**158 (18.2)**	**128 (14.8)**	**89 (10.3)**	**867 (100)**
Median (IQR)^*^^,^ ^**^	56 (24.9)%^#^^,^ ^@^^,^ ^&^	16.2 (6.7)%^#^	12.8 (6)%^@^	12.1 (6.6)%^&^	NA

*Percentages add across. IQR, interquartile range*.

* *Median (IQR) of the proportions of cases per quarter*.

** *Kruskal-Wallis test for significance of the difference between the medians: χ^2^ = 18.5, p < 0.001*.

Significant differences between medians are indicated by similar symbols:

# *p = 0.001*,

@ p < 0.001, and

& *p < 0.001*.

*NA, not applicable*.

The proportion of cases varied from 37 to 82% in the 1st quarter, 8–30% in the 2nd quarter, 6–22% in the 3rd quarter, and 11–33% in the 4th quarter (Table 4). The median and IQR of the proportions of confirmed cases in each quarter is shown in Table 4. The median proportion of the first quarter was significantly higher than that of the second (*p* < 0.001), third (*p* < 0.001), and fourth quarters (*p* < 0.001). The difference between the third and fourth quarter medians approached significance (*p* = 0.053) whereas the difference between the second and third quarter medians (*p* = 0.579), and that between the second and fourth quarter medians (*p* = 0.182), were not significant. Used as a “crude” index of the measure of seasonality of LF, the proportion of cases in the first quarter was <50% in 6 of the 10 years, 2009–12, 2014, and 2017 (Table 4).

The quarterly variation in the proportion of cases of LF admitted to the LFW from 2011 to 18 is shown in Table 5 and illustrated in Figure 5. The proportion was highest in the 1st quarter from 2011 to 18 (35–76%) except in 2014 when it was highest in the 2nd quarter (42%) (Table 5 and Figure 5). The median and IQR of the proportion of admissions in each quarter is shown in Table 5. The median of the first quarter was significantly higher than that of the second (*p* = 0.001), third (*p* < 0.001), and fourth quarters (*p* < 0.001) whereas the differences between the others were not significant (*p* = 0.105 for 2nd vs. 3rd quarters; *p* = 0.189 for 2nd vs. 4th quarters; and *p* = 1.000 for 3rd vs. 4th quarters). Used again as a “crude” index of the measure of seasonality of LF, the proportion of cases in the 1st quarter was ≤50% in 4 of the 8 years, 2011–12, 2014, and 2017 (Table 5 and Figure 5).

### Hospital Case Fatality Among Patients With LF, 2001-18 and 2001-9 vs. 2011–18

**Figure 6:**
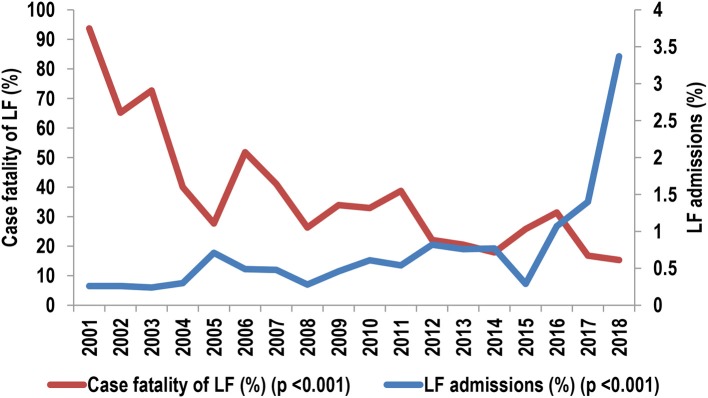
Case fatality of LF vs. the contribution of LF to admissions, 2001–2018.

Table 6 shows the variations in case fatality (CF) among patients admitted with LF from 2001–18 while Figure 6 illustrates the trend; both Table 6 and Figure 6 also show the contribution of LF to admissions during the period for ease of comparison. The trend in CF among patients with LF showed 2 phases: the first was that of a sharp decline from 2001 to 05, while the second was that of irregular increases followed by decreases over a period of generally steady gradual decline from 2006 to 18 (Table 6 and Figure 6). The trend in contribution to admissions also showed 2 phases, but these were the opposite of the trend in CF: an initial phase of gradual increase with irregular periods of alternating increases and decreases from 2001 to 15 followed by a period of sharp increases from 2016 to 18 (Table 6 and Figure 6).

**Table 6:** Contribution of LF to admissions vs. case fatality of LF, 2001–2018.

**Year**	**No. with LF/total no. of admissions (%)**	**No. of deaths/total no. of patients with LF (%)**
2001	16/6,150 (0.23)	15/16 (93.7)
2002	23/8,850 (0.23)	15/23 (65.2)
2003	22/9,092 (0.2)	16/22 (72.7)
2004	25/8,388 (0.3)	10/25 (40)
2005	65/9,215 (0.7)	18/65 (27.7)
2006	54/11,060 (0.5)	28/54 (51.8)
2007	56/11,644 (0.5)	23/56 (41.1)
2008	38/13,617 (0.3)	10/38 (26.3)
2009	56/12,292 (0.5)	19/56 (33.9)
2010	76/12,374 (0.6)	25/76 (32.9)
2011	62/11,537 (0.5)	24/62 (38.7)
2012	113/13,589 (0.8)	25/113 (22.1)
2013	83/10,969 (0.8)	17/83 (20.5)
2014	67/8,671 (0.8)	12/67 (17.9)
2015	31/10,793 (0.3)	8/31 (25.8)
2016	102/9,566 (1.1)	32/102 (31.4)
2017	161/11,537 (1.4)	27/161 (16.8)
2018	248/7,363 (3.4)	38/248 (15.3)
Total	1298/185,707 (0.7)	362/1,298 (27.9)
p^*^	<0.001	<0.001

* *χ^2^ test*.

The averaged CF in 2001–2009 was 154/355 or 43.4% (range = 33.9–93.7%) while that in 2011–2018 was 183/867 or 21.1% (range = 15.3–38.7%). The averaged CF was significantly higher in 2001–09 (OR [95% CI] = 2.9 [2.2, 3.7], *p* < 0.001). The mean ± standard deviation of CF in 2001–2009 (50.3 ± 22.8%) was also significantly higher (*p* = 0.007) than that of 2011–2018 (23.6 ± 8.1%). We excluded the admissions in 2010 from this analysis because some were in the “general” wards (CF = 24/70, 34.3%) and some in the LFW (CF = 1/6, 16.7%; *p* = 0.072 for the difference).

### Review of Factors Associated With Case Fatality Among Hospitalized Patients With LF

We reviewed the factors associated with outcome in four observational studies from ISTH, two published, one in press, and one unpublished but under review for publication; two involved adults, one involved children, and one pregnant women. The presence of shock, bleeding, encephalopathy (as evidenced by seizures and/or coma), and acute kidney injury (AKI) were the major determinants of outcome. Presentation with abnormal bleeding, and ≥1 feature of AKI was associated with increased case fatality in all four studies while encephalopathy was associated with increased case fatality in three of the four (Table 7). Presentation with ≥2 of these complications or danger signs would be equivalent to a SOFA score of ≥2 (35) as criteria for diagnosis of severe infections.

**Table 7:** Factors associated with case fatality in four observation studies at ISTH, Irrua.

	**Adults**		**Children**	**Pregnant women**
References	Asogun et al. (22)	Okokhere et al. (25)	Akpede et al.^#^	Okogbenin et al. (31)^*^
Period reviewed	2009–2010	2011–2015	2009–2017	2009–2018
No. of patients with known outcome	198	284	57	30
No. (%) died	61 (30.8)	68 (24)	16 (28.1)	11 (36.7)
Factors associated with death (^**^Odds ratio [95% CI])				
Abnormal bleeding	Yes (6.2 [2.1, 18.2])	Yes (1.9 [1.1,3.4])	Yes (17.7 [4.4, 71.3])	Yes (not applicable^@^)
Shock	ND	No	Yes (30.8 [3.4, 285.4])	ND
Acute kidney injury	Yes^**^ (ND)	Yes (15 [8,28])	Yes (29.6 [3.2, 275.7])	Yes (31.5 [3, 333.2)
Encephalopathy^&^	No (2.9 [0.8,10.6])	Yes (15 [7,34])	Yes (15.6 [4.2, 72.8)	Yes (31.5 [3, 333.2])

# *Unpublished personal communications (under review for publication); ND, no data*.

* *The same factors were associated with both maternal death and fetal loss; a non-viable pregnancy (factor not included in the table) was associated with OR (95% CI) for death of 17.1 (1.8, 163.8)*.

** *Odds Ratio [95% Confidence Intervals] of death vs. survival*.

@ *9/11 with vs. 0/19 without extra-vaginal bleeding died*.

** *Data not available on the numbers with acute kidney injury but both the mean blood urea nitrogen (p < 0.001) and mean serum creatinine (p < 0.001) were significantly higher among those who died compared with those that survived*.

& *Defined by the presence of coma and/or seizures*.

### Origin/Sources of the Patients in the Study, 2008–18

As with the data on annual variations in numbers of suspected and confirmed cases of LF, data for 2001–08 was not available because we lacked facilities for laboratory diagnosis before 2008 and data availability is limited to Edo State only for the period. A total of 10,367 (73.2%) of the 14,168 suspected cases investigated between 2008 and 2018 were from Edo State while 3,801 (26.8%) were from other states/FCT and Abuja. The corresponding figures for confirmed cases (*n* = 1637) were 957 (58.5%) and 680 (41.5%). The test output among suspected cases from Edo State (957/10,367; 9.2%) was significantly lower than that from other states (680/3,801; 17.9%) (OR [95% CI] of test output from Edo State vs. other states = 0.5 [0.4, 0.5], *p* < 0.001).

**Figure 7:**
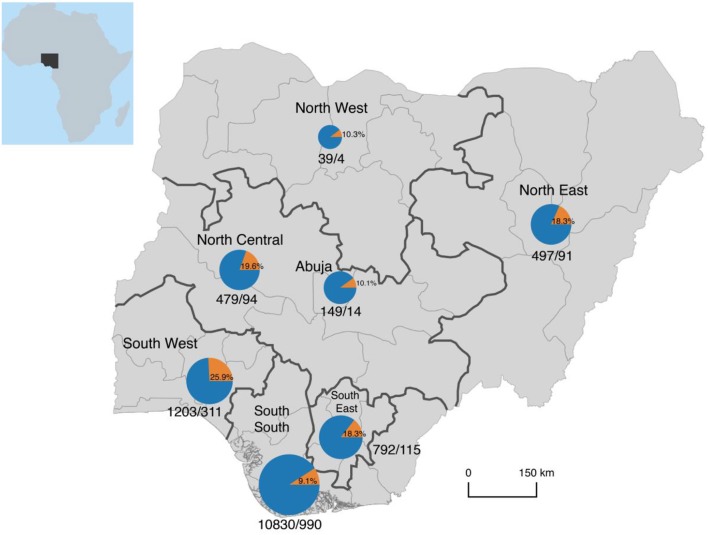
Geopolitical origin of persons with suspected and confirmed Lassa fever as seen at Irrua Specialist Teaching Hospital, Nigeria, 2008-18. Please note that the area of the pie charts is scaled logarithmically to the total number of cases, otherwise the chart for the South-South region would dwarf all others in size. Inset = Map of Africa showing the location of Nigeria.

The geopolitical origins of suspected and confirmed cases are shown in Table 8 and illustrated in Figure 7. South-South Zone had the largest proportion of suspected cases, 76.4 vs. 23.6% from the other five zones. The zone also had the largest proportion of confirmed cases, 60.5 vs.39.5% from the other zones. Within the South-South Zone, Edo State had 95.7% of suspected and 99.7% of confirmed cases. Outside the South-South Zone, Northwest had the lowest proportion of both suspected and confirmed cases (0.3 and 0.2%, respectively) while Southwest had the highest (8.5 and 19%, respectively).

**Table 8:** Geopolitical origins of patients with suspected and confirmed LF as seen at ISTH, 2008–18.

**Geopolitical zone of origin**	**Suspected cases**			**Total no. of states in the zone**	**No. of states with confirmed cases**
	**Total no. (%)^@^**	No. (%) with confirmed infections^#^^,^^*^	**% of confirmed infections^@^**		
South-South	10,830 (76.4)	990 (9.1)	60.5	6	3
South-East	792 (5.6)	115 (14.5)	11.6	5	3
South-West	1,203 (8.5)	311 (25.9)	19	6	1
North-East	497 (3.5)	91 (18.3)	9.2	6	4
North-West	39 (0.3)	4 (10.3)	0.2	7	3
North-Central	479 (3.4)	94 (19.6)	5.7	6	5
Abuja/FCT	149 (1)	15 (10.1)	0.9	NA	NA
Uncertain	179 (1.3)	17 (9.5)	1.0	NA	NA
Total	**14,168 (100)**	**1,637 (11.6)**	**100**	**36** **+** **Abuja/FCT**	**19** **+** **Abuja/FCT**
Edo State	10,367 (74.1)	957 (9.2)	59.1	1	1
Other SS States	463 (3.3)	33 (7.1)	2	5	2
Non-SS States	3,159 (22.6)	630 (19.9)	38.9	30 + Abuja/FCT	16 + Abuja/FCT

*NA, not applicable. FCT, Federal Capital Territory. SS, South-South geopolitical zone*.

@ *Percentages add downwards please*;

# *percentages add across please*.

* *p < 0.001 (χ^2^ test)*.

## Discussion

We set out in this paper to review our experience of the contribution of LF to admissions and mortality among in-patients, and to determine the trend in outcome of hospitalized cases. The results show more fully the spread of LF in Nigeria, the variations in intensity of the outbreaks in affected areas, and the blurring of its seasonality. The results also show the continuing decline in case fatality in contrast to the increasing caseload. Furthermore, the results of mini meta-analysis of determinants of outcome in our center show the feasibility of classification of case severity using exclusively clinical indices. We discuss these and other observations in the study.

### Caseload of LF

We assessed the caseload of LF using the three inter-related indices of number of confirmed cases, contribution to admissions, and contribution to mortality among in-patients. There was a correlation between the trends of all three indices although the contribution to mortality was in excess of and disproportionate to the contribution to admissions throughout the period. Thus, while the contribution to admissions increased from 0.3% in 2001 to 3.4% in 2018, the contribution to deaths rose from 1.5 to 8.8% over the same period. In keeping with the greater endemicity of LF in Sierra Leone, the contribution of LF to admissions and deaths in this study is lower than that reported from Sierra Leone in the 1980s when 10–16% of adult medical admissions and 30% of deaths among hospitalized adults were due to LF (5). Recently published data on the contribution of LF to deaths among in-patients in Sierra Leone are not available but recent reports indicate that the endemicity of LF in the country has remained high, and might indeed have increased (11). The CF among hospitalized patients with LF has also increased above the pre-conflict levels, which were lower than those in Nigeria (6). Thus, 11% of patients who met the case definition for LF in a study from 2008–12 were LASV antigenemic and 23% of non-antigenemic patients had anti-LASV serum IgM antibodies while 10 of the 13 districts in the country had at least one confirmed case of LF (11). CF was 49% in the combined population of antigenemic and non-antigenemic patients compared with a CF of 8–38% pre-conflict (11).

There was a major surge in caseload in 2018, which began earlier in 2016 and before which there were lesser irregular surges in 2005, and 2012–2014. The trend demonstrates the steadily increasing burden of LF in Nigeria and neighboring countries (4) in the absence of effective control measures (2). The results of recent studies, which show that increased rodent-to-human transmission may be a major factor in the recent surges (36–38) suggests that anti-rodent measures and other measures designed to reduce rodent-to-human transmission should constitute a major thrust of the control measures in the country and in the sub region, if the trend is to be stemmed and/or reversed.

It was required to disaggregate the trend in Edo State from that observed with respect to the other states for two main reasons. First, much is already known about LF outbreaks in Edo State (22, 25–27, 39), which remains the epicenter of outbreaks in the country (40, 41). Second, it seemed reasonable that the data on the contribution of LF to admissions and deaths should be from the established diagnosis and treatment center in the state, which was the only such center in the country until recently. We believe that the results, including the differences in test output and caseload between Edo and other states, justify the disaggregation.

The relative preponderance of both suspected and confirmed cases from Edo State decreased over the years, from 2008 to 2018, although the reduction in the proportion of confirmed cases was more from 2012. Thus, the proportion of suspected cases decreased from 89.4% in 2008 to 59.4% in 2018 and that of confirmed cases from 85.7 to 51.5%. The difference between Edo State and others might be because of greater public and health care workers' awareness owing to the longer standing and perhaps more intense efforts in enlightenment/awareness campaigns spearheaded by ISTH and its ILFR&C. The trend gives hope that investments in public awareness campaigns and capacity building of healthcare workers toward raising the index of clinical suspicion for the purpose of engendering early diagnosis and treatment can lead to reductions in caseload and improved outcome over time.

It is possible that the trend in test output is a reflection of the sampling efforts, which could be uneven between different years, and different geopolitical regions but this position is not supported by the available data. The trend is evident both with regard to the absolute numbers of confirmed cases and with regard to the test output from suspected cases, which should be capable of correcting for whatever bias there might be in the use of absolute numbers only. In addition, the trends both countrywide and in Edo State were similar; Edo State accounted for about 40% of the cases seen nationally during the 2018 and 2019 outbreaks (41), and might have accounted for much higher proportions in earlier outbreaks.

### Seasonality of LF

We have in earlier reports (22, 39) drawn attention to the blurring of the seasonality of LF in Nigeria, and similar observations have been reported from Sierra Leone (11, 21). The results of this study, which covers a much longer period, confirms earlier observations (11, 21, 22, 39). The transition might be due to many factors including population displacements due to perennial flooding, perhaps due to the famed problem of global warming (42, 43), and the increasing frequency and severity of civil unrest and insurgency (44). There are also the growing problems of poverty (45), slum dwellings and poor management of solid wastes in the Nigeria. These coupled with the now regular burning of bushes, might be hitherto unacknowledged factors associated with increased opportunities for rodent-to-human transmission of infection. In addition, variations between geographic regions and the risk of LF in West Africa has been associated with variations in annual rainfall (18) and climatic changes due to global warming (42, 43) could therefore also by itself be a factor in the change in seasonality. Irrespective of the factors involved, however, the change in seasonality makes outbreak preparedness and response an all year-round necessity.

The need for all-year round preparedness is buttressed by the findings that although the proportion of cases of LF was highest overall (59.9% of the annual prevalence) in the 1st quarter from 2008 to 18, <50% of the annual prevalence was in the 1st quarter in six (2009–12, 2014, and 2017) of the 10 years. In addition, 27.6% of the combined annual prevalence, range = 14.5% in 2016-47.2% in 2017, occurred during the raining season period (2nd and 3rd quarters). Shaffer et al. (11) in Sierra has also observed the occurrence of a second peak of LASV antigenemia during the rainy season, the other peak being in the dry season.

### Case Fatality of LF

The trend in case fatality of LF contrasts sharply with the trends of the contribution of LF to admissions and deaths among in-patients. These contrasts emphasize the place of deployment of effective medical response in the control of outbreaks, to give hope in place of despair. The occasional and irregular surges in case fatality in 2003, 2006, and 2016 were most probably due to acute shortages of ribavirin, which is unfortunately recurrent (2, 46). There might also have been occasions of an increase in case severity, particularly in 2016 (47), which are not readily explained. Increased virulence, perhaps due to the emergence of a new lineage or sub-lineage to which there might have been low herd immunity was one of the explanations advanced earlier for the apparent increase in case severity in 2016 (47) but this explanation is not supported by the results of genomic analysis of the 2018 surge in LF outbreak in Nigeria which showed that the increased caseload was more likely due to increased rodent-to-human transmission and not the appearance of new lineages (36–38).

Other studies on CF during the 2016 outbreak in Nigeria have reported varied figures: Buba et al. (48) reported a CF of 59.6% among 47 confirmed cases, and noted that CF was up to 77.8% in some states; Shehu et al. (49) reported a CF of 36% among 11 confirmed cases and noted that this was lower than the 83% from an earlier study in 2012 in the same state; and the European Centre for Disease Prevention and Control (ECDC) reported a CF of 60% among 57 confirmed cases (4). The CFs reported by Buba et al. (48) and ECDC (4) are about double the CF of 31.4% in our series in 2016, a year during which there were shortages of ribavirin, while the CF of 36% reported by Shehu et al. (49) in 2016 is not markedly different from ours. Neither Buba et al. (48) or ECDC (4) attempted to explain the high CFs while Shehu et al. (49) thought that the much higher CF in 2012 was due to late presentation, low index of suspicion among clinicians and lack of access to ribavirin.

Early diagnosis and treatment, engendered by regular public awareness campaigns and capacity building of health care workers toward the development of a high index of clinical suspicion, as well as the build up of experience in clinical case management, might be factors in the steady reductions in case fatality at our center. The ready availability of diagnostic facilities locally since 2008 (22), which facilitates early diagnosis, would clearly also be an important factor. There are also the on-going improvements in treatment facilities at the center, particularly since the commissioning of the LFW. For example, dedicated facilities for hemodialysis of infected patients with AKI became available in 2011 and facilities for respiratory support toward the end of the surge in outbreak in 2018 while the facilities for component blood therapies are under development.

It is however, notable that there were already marked decreases in CF before the establishment of the LFW and that the rate of reduction after its commissioning was lower than expected. In relation to the first observation, it is possible that the more severely ill patients were assessed and treated for LF in the days before establishment of the Institute and availability of diagnostic facilities. This situation, which could be associated with a low index of suspicion, low levels of awareness and late presentation, acting in conjunction with the frequent lack of ribavirin and limited capacity for supportive care, would not surprisingly be associated with high CFs as in the first few years covered by this report. As awareness and index of suspicion increased, and there was earlier presentation and diagnosis/institution of treatment, decreases in CF could be expected. This might also partly explain the sharp drops in CF in the earlier part of the study period. Furthermore, the practically of onsite diagnosis, and potential for early diagnosis, following the establishment of the D&RL before the coming on board of the LFW may also have contributed to the further declines in CF in the initial part of the study.

The apparent lack of a marked impact of establishment of the LFW on the trend in CF could be due to several factors: *First*, marked improvements in clinical case management experience might have predated its establishment. *Second*, it could be that the impact of treatment with ribavirin has approached saturation. *Third*, the supportive clinical care facilities, other than those for hemodialysis, are still at an early stage of development, and it should be reasonable to expect that the availability of more comprehensive and specialized care could result in further improvements. Thus, we have had patients who might have died if the provision of hemodialysis for AKI or assisted ventilation for respiratory failure was unavailable. These factors taken together could mean that further significant reductions in case fatality might not be forthcoming without the establishment of full facilities for enhanced supportive or critical care and/or the development of pharmacologic therapies that are synergistic with or alternative to ribavirin (50–52). However, although this underlies the need for acceleration of the development of additional treatment resources, it would also not be unreasonable to further explore public health measures directed at further addressing delays in presentation. Most of the cases originate at the community level and a rigorous exercise of community engagement coupled with active community-based surveillance could address the problems of late presentation and case detection. This requires deliberate efforts in building the capacity of volunteers at the community level.

The lack of results from this study that are strongly supportive of the impact of dedicated treatment facilities on CF notwithstanding, we strongly believe that the pooling of human and material resources for clinical case management of severely ill patients with LF and other severe VHFs could greatly impact on the hospital outcome. Although there was no dramatic decline in CF after establishment of the LFW, it is still nonetheless notable that the trend of a steady decline in CF was sustained. For emphasis perhaps, the expectation of further rapid reduction could have remained unmet because the process of provision of facilities for enhanced supportive/critical or intensive care for severely ill patients has been protracted. We can attest from our experience in the treatment of severe LF (25, 53, 54), and from the experience of others in the treatment of severe VHFs such as Ebola both overseas and in the sub region (16, 55–57), to the benefits of such provisions as hemodialysis and respiratory support among others. We yet lack adequate facilities for the treatment of patients with encephalopathy, as well as facilities for provision of adequate cardiovascular support while those for hematologic support are under development and facilities for blood gas determination, *et cetera*, are awaited. Also, the provision of point-of-care facilities for radiologic, microbiologic, and even clinical chemistry assessment has remained a challenge. And, even though hemodialysis has been available for some time, it is expensive and many patients are not able to meet the cost. We anticipate that it should be possible to more fully assess the impact of the dedicated diagnosis and treatment facility in years to come when these provisions have been made.

Thus, we believe that the establishment of dedicated, strategically located treatment centers can help to address major aspects of the clinical challenge of LF, particularly the (currently) unacceptably high case fatality in endemic areas (6, 11, 22, 25, 48, 49, 58). Adequate treatment is crucial to reduction of case fatality in severe VHFs (15, 16) and the establishment of dedicated centers and triaging of patients for referral, working in tandem can enable the pooling of personnel and facilities for the optimization of treatment approaches in reducing the risk of death. Therefore, we propose that treatment centers, preferably coupled with surveillance centers, be established in strategic “zones” in endemic areas as “hubs” with “spokes” drawn from surrounding health facilities in a “hub and spoke” referral system. For example, Nigeria with her 6 geopolitical zones could have one center per zone. The centers should be capable of accommodating surges in caseload and illness severity and could also serve the clinical care needs of patients with other severe VHFs. We are gratified to note that drawing on our experience, the strategic establishment of dedicated treatment centers in Nigeria has commenced as part of the national medical response to LF. And, it could clearly be quite helpful to compare the experience of the new centers with ours in future.

To be effective, these centers should have capacity for enhanced supportive/critical care (59–62) for the goal-directed treatment of patients with severe infections. This is because the pathophysiologic changes in severe VHFs (59, 63, 64) are akin to those of severe sepsis/septic shock (65) and should be addressed accordingly (59, 64). Septic shock, less commonly hypovolemic shock from fluid loss or severe bleeding, AKI, and respiratory failure are the common complications associated with death in severe sepsis (35, 66, 67), including severe VHFs such as Ebola (35, 65, 68) and LF (22, 25).

We should also emphasize the added medical and public health advantages in having dedicated surveillance and treatment centers. None of the staff in the ILFR&C and its LFW has taken ill with LF since establishment of the LFW in 2010. On the other hand there have been regular reports of infection of healthcare workers (HCW) from other places (29, 40, 69). For example, among 381 confirmed cases of LF from 21 states reported between January 1st and February 24th 2019, 15 (3.9%) were infected HCWs (70), and 45 (7.1%) out of 633 confirmed cases between January 1st and December 31st in 2018 were also HCWs (71). Mustapha (72) has detailed the incidence of infection of HCW with LF in recent years in an editorial aptly titled “Lassa fever: Unveiling the misery of the Nigerian health worker”; “as at January 28, 2018” for example, 10 (13%) of 77 confirmed cases of LF were among HCWs and 4 of the 10 died (72).

The trend in CF and the factors associated with outcome among our patients also merit discussion from another perspective, which could be a further pointer to some of the actions required in improving the outcome of severe LF/other severe VHFs in West African. The association between mortality and presentation with ≥2 of four clinical features including abnormal bleeding, shock, indices of AKI, and features of encephalopathy, was crosscutting among the various demographic groups of patients with LF (22, 25, 31, Akpede et al., personal communication). This could form the basis for the classification of patients and choice of treatment pathway on presentation. We suggest that the presence of ≥2 risk factors could be taken as indicative of severe infections and the need for referral to higher levels of care while patients with mild infections (absence of a major risk factor) are managed locally. Operational research is however, required to validate this recommendation.

### Other Issues

There are at least three other issues arising from or during this study that should be discussed. *First*, at least two ribavirin dosage regimens in the treatment of LF are in use in Nigeria. The first entails the administration of single or once daily doses (22, 28, 29) while the second entails the administration of multiple daily doses (12, 13, 30, 32). Beside this difference, the total dose of ribavirin administered over the course of treatment in the first regimen is lower than that in the second. The first regimen might theoretically be attended with fewer side effects, lower risk of nosocomial transmission of infection, and lower cost of care. However, there have as yet been no formal comparative analysis of the associated course and outcomes of illness associated with the two regimens, but anecdotal observations suggest that they might be comparable. Further studies are required to guide recommendations on the optimal dose and dosage of ribavirin, and could also be useful in addressing the growing concern with treatment costs (73).

*Second*, the costs of diagnosis and treatment might become major issues in access to care, with implications for the sustainability of surveillance and treatment programs. The dependence on overseas partners is becoming more strenuous and exerting. It is clearly not sustainable, and calls for urgency in the development of more affordable reagents and diagnostic alternatives. It also calls for the development of indigenous methods of diagnosis that do not depend on the importation of all component reagents from overseas.

Reducing the cost of care is of a growing concern (73), and is an important issue with the dependence on out-of-pocket expenditure as a major source of healthcare financing in many developing countries (74, 75). The Federal Government of Nigeria has been subsidizing the clinical care of infected persons but this has sometimes been plagued unfortunately by recurrent and severe shortages of ribavirin (46), with serious implications. A sense of urgency in the development of less expensive and preferably sub regionally sourced pharmaceutical products is desirable.

The issue of cost of treatment raises other concerns. For example, do all confirmed symptomatic cases require the same care as is currently the practice of a one-size-fits-all approach? Do they all even need hospitalization, intravenous fluids, antibiotics, and antimalarial drugs? Do they all require ribavirin, and if they do, should it be intravenous all the time, and for how many days really? And, is protection with a full set of standard personal protective equipment required to reduce the exposure of healthcare workers to nosocomial transmission in all cases? Recovery from symptomatic infections without treatment with ribavirin is possible (76) and the risk of transmission of infection from severely ill patients to health care workers can be considerably reduced, with the use of standard precautions (77–79). Furthermore, the open trial of ribavirin by McCormick et al. (12) included severe cases of LF which were treated with oral ribavirin and did significantly better than those on placebo [It should be acknowledged however, that there was a clear survival advantage with the use of intravenous ribavirin (12)]. Thus, the answers to some, if not all the questions might probably be no. Operational research is urgently needed to address these and other issues, and provide the required evidence for a review of extant and current treatment guidelines toward modulating the cost of care. It is probably also necessary to redefine the threshold for clinically significant illness in terms of clinical plus/minus laboratory indices.

*Third*, the prevalence of LF of about 12% among patients with suspected infections in 2008–2018 is higher than the 6% reported from among a small group (*n* = 31) of febrile patients evaluated in 2003–2004 (26) but lower than the 42% reported from a larger group (*n* = 60) of patients investigated while on admission with “suspected Lassa fever” in 2005–2008 (27). In the latter study (27), LF was suspected in the presence of negative laboratory investigations for malaria and bacterial infections or persistence of fever despite treatment with antimalarial and antibiotic drugs or presentation with known signs of LF (27). Although the criteria are like some of those used in the current study, there might have been an over-selection of severely ill patients who were more likely to be positive for LF. In contrast, the criteria used in the selection of patients in the current study were more robust. In addition, the patients involved in this study were recruited from all settings in the hospital, and not limited to those on admission as in the earlier study (27). The test output in our study might thus be more representative of the true prevalence of LF among febrile patients seen in endemic areas in Nigeria. The increase in prevalence over that reported from the earlier study (26) might be reflective of a true increase in the prevalence of LF rather than being simply a reflection of improvement in diagnostic capacity and increased clinical index of suspicion.

Ilori et al. (41) reported a decline in test output among suspected cases from 31% during the peak of the 2017 outbreak to 21.6% in 2018. In contrast, there was an increase from 12.8% in 2017 to 17.8% in 2018 among our patients. The decrease reported by Ilori et al. (41) is difficult to explain against the background that the 2018 outbreak was the largest reported so far from Nigeria. On the other hand, the lower test output in our study could be because Ilori et al.'s report (41) was in relation to the peak of the outbreaks in 2017 and 2018 whereas ours represented yearly averages.

The trends in test output and caseload further strengthens the general case for the screening of febrile patients in endemic areas for LF (2, 26, 32) and thus the imperative of accelerating the development of rapid diagnostic tests/point of care tests for the diagnosis of LF, and other VHFs in endemic areas. The trends also underlie the need for a sense of urgency in scaling up the institution of general control measures and the further development of medical counter measures as advocated by the WHO/Welcome Trust (80). LF has the potential to frustrate attainment of the sustainable development goals in West African countries.

## Limitations of the Study

There at least two limitations inherent in this study, which should be discussed. *First*, the LF case fatality component of the study was restricted to patients with RT-PCR-confirmed infections whereas some patients might have died at the emergency units of the hospital (Accident and Emergency Unit, Children Emergency Ward, Labor Ward) with unconfirmed infections. In addition, none of the confirmatory tests used in the study has 100% sensitivity (81). Thus, the estimates of the contribution of confirmed cases to admissions and deaths as well as the estimates of caseload and case fatality of LF could be different from that reported in this paper. However, we have no reasons to think that the differences are substantial.

*Second*, a detailed analysis of the factors associated with hospital outcome of cases of LF was not done, partly because it was beyond the scope of the study and also because it was the subject of other studies (22, 25, 31, Akpede et al., personal communication). Furthermore, the lack of uniformity in the standard of care during the period covered by the report would have made it quite difficult controlling for the many potential confounding factors that might feature owing to the variation. Despite this, however, we believe that the results give at least a broad view of the trend in outcome over the years.

## Conclusion and Recommendations

This is the first “comprehensive” report on LF in Nigeria that assessed the trends for at least a decade and inter-related the spatial and temporal aspects of the outbreaks. It shows that LF has strengthened both spatially and temporally, with the potential for cycles of outbreaks of increasing severity. The report should serve to reduce the level of uncertainty regarding the spatial and temporal epidemiology of LF in Nigeria and further calls for urgent efforts in reinvigorating control measures and reinvigorating the search for sustainable solutions (2).

In addition, the study demonstrates the gains of a sustained trend in the reduction of LF case fatality, which might be related to the establishment of a dedicated clinical case management center. Thus, replication of the establishment of “hubs” of LF surveillance and treatment centers could be a pragmatic and cost-effective approach in rising to the clinical challenge of LF in endemic countries.

## Author Contributions

GA, DAA, SO, CH, PS, DEA, MR, SG, MM, and AD: conceptualization of study. NA, GO, EM, FO, OO, CO, and CIk: data retrieval. GA and AC data analysis. GA: initial draft of manuscript. GA, EO-E, DAA, SG, MR, CH, AC, CIk, and CIh: revision of draft manuscript. GA, SD, MO, RA, AA, FO, AD, MM, and SO: clinical care of the patients. IO, DIA, and EM: laboratory work. GA, ET, DAA, SO, SD, MM, AD, CIh, NA, and EO-E: administration. SG, CH, PS, DAA, SO, GA, and CIh: securing funding for the project.

### Conflict of Interest Statement

The authors declare that the research was conducted in the absence of any commercial or financial relationships that could be construed as a potential conflict of interest.
