# Corrigendum: Caseload and Case Fatality of Lassa Fever in Nigeria, 2001–2018: A Specialist Center's Experience and Its Implications

**DOI:** 10.3389/fpubh.2019.00251

**Published:** 2019-09-13

**Authors:** George O. Akpede, Danny A. Asogun, Sylvanus A. Okogbenin, Simeon O. Dawodu, Mojeed O. Momoh, Andrew E. Dongo, Chiedozie Ike, Ekaete Tobin, Nosa Akpede, Ephraim Ogbaini-Emovon, Adetunji E. Adewale, Oboratare Ochei, Frank Onyeke, Martha O. Okonofua, Rebecca O. Atafo, Ikponmwosa Odia, Donatus I. Adomeh, George Odigie, Caroline Ogbeifun, Ekene Muoebonam, Chikwe Ihekweazu, Michael Ramharter, Andres Colubri, Pardis C. Sabeti, Christian T. Happi, Stephan Günther, Dennis E. Agbonlahor

**Affiliations:** ^1^Institute of Lassa Fever Research and Control, Irrua Specialist Teaching Hospital, Irrua, Nigeria; ^2^Department of Paediatrics, Irrua Specialist Teaching Hospital, Irrua, Nigeria; ^3^Department of Community Medicine, Irrua Specialist Teaching Hospital, Irrua, Nigeria; ^4^Department of Obstetrics and Gynaecology, Irrua Specialist Teaching Hospital, Irrua, Nigeria; ^5^Department of Surgery, Irrua Specialist Teaching Hospital, Irrua, Nigeria; ^6^Department of Nursing Services, Irrua Specialist Teaching Hospital, Irrua, Nigeria; ^7^Department of Medical Records, Irrua Specialist Teaching Hospital, Irrua, Nigeria; ^8^Nigeria Centre for Disease Control, Abuja, Nigeria; ^9^Department of Tropical Medicine, Bernhard Nocht Institute for Tropical Medicine & I. Department of Medicine University Medical Center Hamburg-Eppendorf, Hamburg, Germany; ^10^Department of Organismic and Evolutionary Biology, Harvard University, Cambridge, MA, United States; ^11^Department of Biological Sciences and African Center of Excellence for Genomics of Infectious Diseases, Redeemer's University, Ede, Nigeria; ^12^Department of Virology, Bernhard Nocht Institute for Tropical Medicine, Hamburg, Germany; ^13^Department of Microbiology, Ambrose Alli University, Ekpoma, Nigeria

**Keywords:** case fatality, caseload, center's experience, implications, Lassa fever, outbreaks, Nigeria, trends

Two author names were incorrectly spelled as “Pardis C. Sarbeti” and “Ekene Muebonam”. The correct spelling is “Pardis C. Sabeti” and “Ekene Muoebonam”.

The authors apologize for this error and state that this does not change the scientific conclusions of the article in any way. The original article has been updated.

